# A Case of Aymé-Gripp Syndromic Congenital Cataracts and Pigmentary Retinopathy Caused by a Novel MAF Variant in the N-Terminal Transactivation Domain—A Case Report and Literature Review

**DOI:** 10.3390/genes17040380

**Published:** 2026-03-27

**Authors:** Max Chauhan, Kaersti L. Rickels, Sudhi P. Kurup, Brenda L. Bohnsack, Alexander Ing, Andy Drackley, Allison Goetsch Weisman, Valerie Allegreti, Kailee Yap, Pamela Rathbun, Andrew Skol, Patrick McMullen, Hantamala Ralay Ranaivo, Jennifer L. Rossen

**Affiliations:** 1 Division of Ophthalmology, Ann & Robert H. Lurie Children’s Hospital of Chicago, Chicago, IL 60611, USA; 2 Feinberg School of Medicine, Northwestern University, Chicago, IL 60611, USA; 3 Department of Ophthalmology, Northwestern University Feinberg School of Medicine, Chicago, IL 60611, USA; 4 Department of Pathology and Laboratory Medicine, Ann & Robert H. Lurie Children’s Hospital of Chicago, Chicago, IL 60611, USAadrackley@luriechildrens.org (A.D.); pmcmullen@luriechildrens.org (P.M.); 5 Department of Pediatrics, Northwestern University Feinberg School of Medicine, Chicago, IL 60611, USA; 6 Edwards Family Division of Genetics & Rare Diseases, Ann & Robert H. Lurie Children’s Hospital of Chicago, Chicago, IL 60611, USA; 7 Department of Pathology, Northwestern University Feinberg School of Medicine, Chicago, IL 60611, USA

**Keywords:** MAF, Aymé-Gripp, congenital cataract, pigmentary retinopathy

## Abstract

*MAF* encodes a transcription factor involved in T-helper-2 (Th2) cell differentiation. Heterozygous pathogenic variants in *MAF* have been observed in both isolated and syndromic congenital cataract cases; genotype–phenotype correlations are based on the location of the variant within the gene. Variants in the N-terminus domain of *MAF* are associated with cataracts as part of Aymé-Gripp syndrome. The purpose of this report is to expand the ocular phenotypic spectrum of Aymé-Gripp syndrome by describing a patient with *MAF* variant c.185C>G, p.Thr62Arg, and the traditional systemic findings and congenital cataracts as well as an unusual feature of pigmentary retinopathy, which has not been previously reported in Aymé-Gripp syndrome. Additionally, a comprehensive review of the literature was completed to report ocular genotype–phenotype data on previously reported patients with *MAF*-associated Aymé-Gripp syndrome.

## 1. Introduction

Aymé-Gripp syndrome is an autosomal dominant condition characterized by a triad of classic symptoms: early bilateral cataracts, sensorineural hearing loss, and characteristic facial features along with neurodevelopmental abnormalities [1,2,3,4,5,6,7,8,9,10,11]. Additional associated features include seizures and pericarditis or pericardial effusion at a young age [1,2]. Other previously reported ocular features include optic nerve anomalies, macular hypoplasia, and retinal hemorrhages and neovascularization; however, retinal degeneration has not been previously reported [4,5,6,9]. The condition is rare, with only 24 individuals described in the literature according to the Human Gene Mutation Database [1,2,3,4,5,6,7,8,9,10,11]. The diagnosis of Aymé-Gripp syndrome is made by identification of a heterozygous pathogenic variant in the N-terminus transactivation domain of the *MAF* gene in individuals with a clinically suspicious phenotype. MAF is a basic leucine zipper transcription factor that is involved in the development of neurologic and ocular structures, bone, skin, kidney, and cochlear cells [3].

This manuscript reports a novel retinal degeneration phenotype in association with congenital cataracts in a patient with Aymé-Gripp syndrome. Additionally, a comprehensive review of *MAF* variants in the N-terminal transactivation domain associated with Aymé-Gripp syndrome was completed with a focus on ophthalmic manifestations.

## 2. Materials and Methods

### 2.1. Patient Information and History

A 5-day old full-term male was referred to the Division of Ophthalmology at Lurie Children’s Hospital for abnormal red reflex in both eyes diagnosed by a pediatrician at birth. His ophthalmologic examination was consistent with bilateral dense nuclear cataracts. There was no reported family history of pediatric cataracts or complications in the pregnancy or delivery. A panel of molecular tests for metabolic diseases and teratogenic maternal infections was found to be negative.

**Figure 1:**
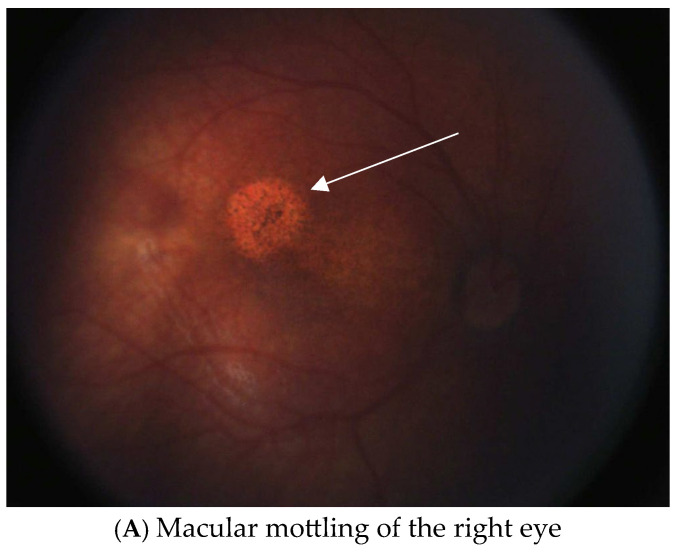

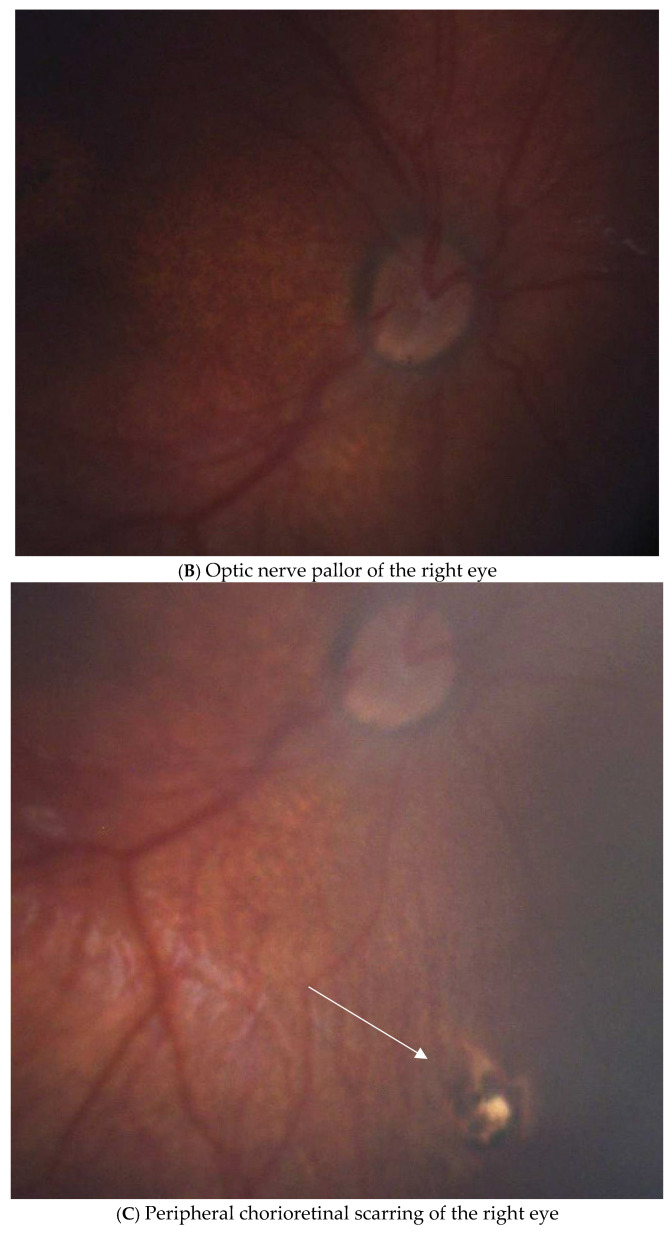

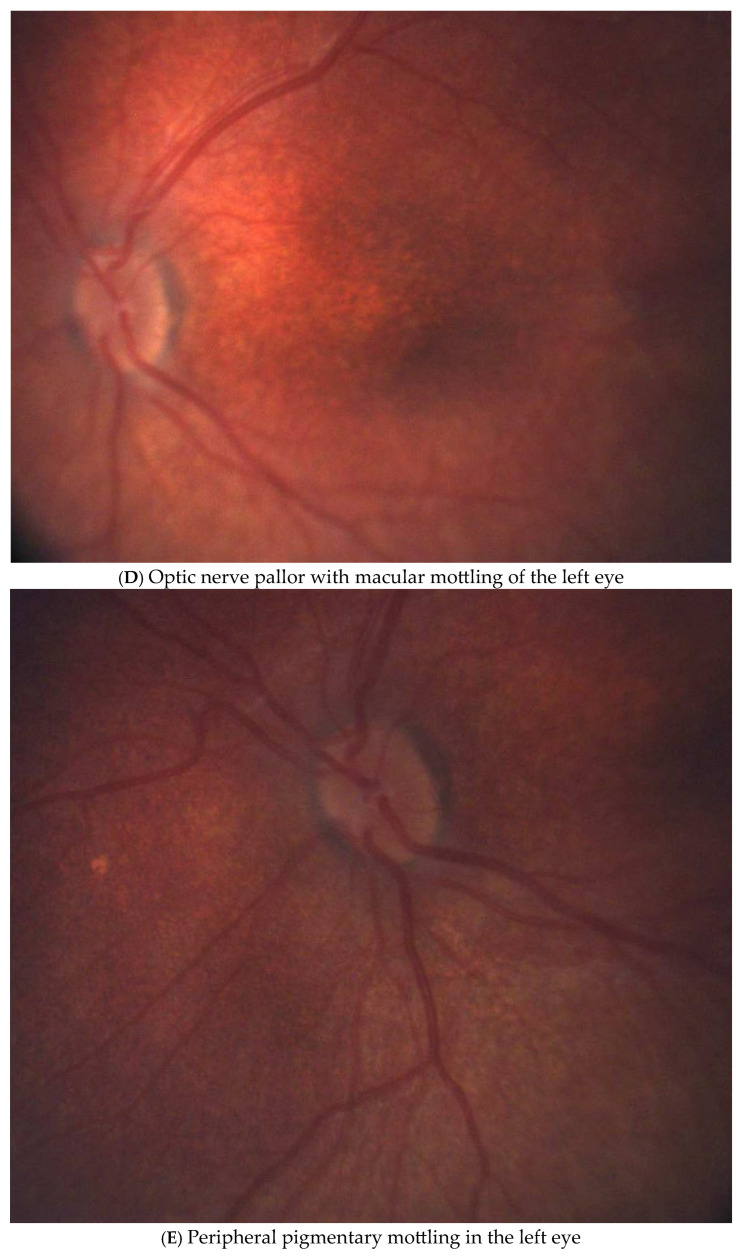
Fundus Photography. (**A**–**E**) include photos of both eyes at 8 months-old exam under anesthesia. Arrows in (**A**,**C**) are pointing to the abnormal retinal findings.

The patient underwent cataract removal surgery of the right eye at 4 weeks and the left eye at 6 weeks old. At 8 months old he was noted to have optic nerve pallor with pigmentary mottling and macular atrophy present in both eyes, along with chorioretinal scarring (Figure 1). In addition to the retinal findings, the proband failed the newborn hearing screen twice, was diagnosed with laryngomalacia and congenital chordee at 1 month old, developed recurrent pericardial effusions requiring drainage at 3 months old, and required G-tube nutrition within the first few months of life. He was closely followed by the Department of Neurology, with a brain MRI noting microcephaly and an incidental finding of Chiari malformation. At 8 months old, an EEG showed risk for the development of seizures, and a regimen of anti-seizure medication was commenced at 1 year old. By 34 months old, the patient’s visual acuity was 20/380 with aphakic contact lenses according to Teller Acuity Cards (TAC).

### 2.2. Genetic Testing

The patient was initially evaluated by a clinical geneticist and genetic counselor at 2 months of age. Due to the multiple medical conditions, the parents were counseled and provided informed consent for the patient to undergo genetic testing via trio-based whole genome sequencing (WGS) with mitochondrial analysis (GenomeXpress through GeneDx, Stamford, CT, USA). Due to the development of retinal findings, the trio WGS data was re-analyzed for variation in genes associated with retinal conditions (GeneDx).

## 3. Results

### 3.1. Genome Sequencing

Trio-based whole genome sequencing identified a de novo heterozygous variant in *MAF*, NM_005360.5:c.185C>G (p.Thr62Arg), which was classified as pathogenic in association with Aymé-Gripp syndrome and not identified in either parent. The support for the pathogenic classification of this variant provided by the lab was: a de novo inheritance pattern, a low frequency in gnomAD, an in silico analysis supporting a deleterious effect on protein structure/function, the presence of previously reported variants in nearby residues, and a previous literature report of the same variant in a patient with Aymé-Gripp syndrome. The only other reported variant was a maternally inherited, heterozygous pathogenic variant in *CFTR* (c.262_263del, p.Leu88Ilefs*22), consistent with carrier status for a CFTR-related disorder. Re-analysis of the trio WGS did not identify any other variants thought to potentially be causative of the patient’s retinal findings. The identified *MAF* variant has been deposited into the public ClinVar database.

### 3.2. Literature Review

A literature review from the Human Gene Mutation Database (HGMD) was completed in January 2025, identifying 24 individuals with *MAF* variants and clinical features of Aymé-Gripp syndrome, to closely examine other reported ocular manifestations (Table 1 and Table 2) [1,2,3,4,5,6,7,8,9,10,11]. Most variants were de novo, like our patient, with three instances of maternal inheritance from a mildly affected parent. All were missense variants in the transactivation domain, as was our patient’s variant. While all patients had cataracts, most reports did not include a description of the cataract type. Additional reported ocular features included eyelid and/or orbital structural abnormalities (n = 17, 85%), optic disc abnormalities (n = 5, 21%), macular hypoplasia (n = 2, 8%), avascular retina with neovascularization (n = 1), retinal hemorrhage (n = 1), and a “white spot on retina” (n = 1). There were no reports of pigmentary retinopathy or retinal degeneration.

## 4. Discussion

MAF is a basic leucine zipper transcription factor that not only is important for eye (and particularly lens) development but also is expressed in many other organ systems including neurologic structures, bone, skin, kidney, and cochlear cells [3]. The 3′ DNA binding domain of *MAF* on the C-terminus contains three functional domains: extended homology, basic motif and leucine zipper regions [3]. The transactivation domain in the N-terminal region is critical for transcription and regulatory function [3]. Within this transactivation domain, there are four highly conserved GSK3 phosphorylation motifs [3]. Most of the reported pathogenic variants associated with are either at these highly conserved phosphorylation motifs or are predicted to affect phosphorylation; this phosphorylation inhibits degradation of MAF and alters the transactivation and regulation of genes on which it acts [3].

The phenotypes associated with variants in one of the C-terminal domains compared to the N-terminus of MAF are distinct. Clinically, pathogenic variation in one of the C-terminus domains is associated with isolated cataracts [3,12] and those in the N-terminal transactivation domain are associated with syndromic cataracts as part of Aymé-Gripp syndrome [3]. Experimental evidence supports this distinction: a zebrafish study demonstrated that patient-identified *MAF* transactivation domain variants in the N-terminus lead to reduced brain development compared to the wild-type and patient-identified *MAF* variants altering the C-terminus [3].

Aymé-Gripp syndrome is a rare condition caused by heterozygous pathogenic variants, usually de novo, in the N-terminus of MAF. In addition to variable, often severe, systemic manifestations, pediatric cataracts are a hallmark feature of this syndrome. The second most common ocular manifestation reported in the literature is lid and orbital anatomical abnormalities that contribute to the often characteristic facial features. Optic nerve anomalies are less commonly reported features. The only reported retinal phenotypes in the literature were a white spot, macular hypoplasia, avascular retina, and retinal hemorrhages but no descriptions of pigmentary mottling or macular atrophy as seen in our patient (Table 2). Our patient was born full-term and other metabolic and infectious causes were ruled out, as were other genetic causes. The other patient in the literature with the same variant was not noted to have retinal findings; however, it is possible that they were not yet identified as the patient was only 21 months old at the time of the report or this could represent variable expressivity of the variant [3]. Ocular manifestations other than cataracts in patients with variants affecting the C-terminus of MAF include microcornea (or more rarely Peter’s anomaly) or iris coloboma without retinal manifestations [12].

Even though *MAF* variants have not been previously reported in association with retinal dystrophies, as seen in our patient, other heterozygous missense variants in residues with the same regulatory motif as other proteins in the MAF subfamily (MAFB and NR) have been associated with autosomal dominant retinitis pigmentosa [3,13,14]. In addition to regulating terminal differentiation of the lens, bone, brain, kidney, and pancreas, MAF proteins have been shown to also regulate the retina, thereby providing support that the *MAF* variant in our patient was likely causative of the retinal findings [3,15,16]. Wang et al. postulated that the macular hypoplasia identified in their patients may have been due to a similar mechanism of *NRL* variants causing retinal pathology [6]. *NRL* is a member of the MAF family and is essential for rod photoreceptor development, and missense variants in the N-terminal transactivation domain (similar to those in *MAF* associated with Aymé-Gripp syndrome) lead to retinitis pigmentosa due to disruption of phosphorylation and translational activity [6]. Especially in light of the negative re-analysis of the WGS to identify another likely cause and the rare reporting of patients with Aymé-Gripp in the literature, we suggest that N-terminus transactivation domain *MAF* variants may be associated with retinal dystrophies in addition to the previously reported lens changes, orbital anatomical differences, and systemic diseases.

## 5. Conclusions

In this paper, we describe a patient with a pathogenic heterozygous variant in *MAF* and a new clinical phenotype of retinal degeneration to expand the ocular phenotype of Aymé-Gripp syndrome. We also review HGMD reported variants in the N-terminus transactivation domain of MAF associated with Aymé-Gripp syndrome and their reported ocular manifestations. Additional cases and functional studies are needed to confirm the relationship of *MAF* variants and retinal degeneration.

## Figures and Tables

**Table 1:** Reported functional variants in the N-terminus of the *MAF* gene associated with Aymé-Gripp syndrome.

Subject ID	Reference	cDNA Variant	Protein Change	Inheritance
19474	[1,3]	c.161C>T	p.Ser54Leu	De Novo
CAGI-UCSC	[3]	c.161C>T	p.Ser54Leu	De Novo, Germline
Individual 1	[4]	c.161C>T	p.Ser54Leu	De Novo
Subject 5	[5]	c.161C>G	p.Ser54Trp	De Novo
Individual 3	[4]	c.170C>T	p.Ser57Phe	De Novo
11-1/pt. 1	[2,3]	c.172A>G	p.Thr58Ala	De Novo, Germline
ID 1	[6]	c.173C>A	p.Thr58Asn	De Novo
ID 2	[6]	c.173C>T	p.Thr58Ile	De Novo
ICN-ICW	[3,7]	c.173C>T	p.Thr58Ile	Unknown
CSA108.01	[8]	c.176C>G	p.Pro59Arg	Maternal
CSA108.02	[8]	c.176C>G	p.Pro59Arg	Unknown
Subject 2	[5]	c.176 C>A	p.Pro59His	Unknown
Subject 3	[5]	c.176 C>A	p.Pro59His	De Novo
14-1/pt. 2	[3,9]	c.176C>A	p.Pro59His	De Novo
10-1	[3]	c.176C>T	p.Pro59Leu	De Novo
Individual 4	[4]	c.176C>T	p.Pro59Leu	De Novo
962112	[3]	c.185C>G	p.Thr62Arg	De Novo
Patient 3	[10]	c.185C>T	p.Thr62Met	De Novo
Subject 1	[5]	c.188C>G	p.Pro63Arg	*Maternal (negative), Paternal not possible*
Subject 4	[5]	c.197C>G	p.Ser66Trp	De Novo
Individual 2	[4]	c.197C>T	p.Ser66Leu	De Novo
4-1/pt. 2	[2,3]	c.206C>G	p.Pro69Arg	De Novo, Germline
Proband	[11]	c.206C>G	p.Pro69Arg	Maternal
Mother	[11]	c.206C>G	p.Pro69Arg	Unknown

**Table 2:** Ocular manifestations associated with Aymé-Gripp syndrome congenital cataracts.

Subject ID	Reference	Cataract Findings	Orbital Findings	Secondary Glaucoma	Optic Nerve Findings	Retinal Findings
19474	[1,3]	Not specified	Hypertelorism, downslanting palpebral fissures	----	----	----
CAGI-UCSC	[3]	Not specified	Downslanting palpebral fissures, hypertelorism, epicanthal folds, palpebral ptosis	----	----	----
Individual 1	[4]	Not specified	Hypertelorism	----	----	----
Subject 5	[5]	Not specified	----	----	----	“White spot on left retina”
Individual 3	[4]	Not specified	Hypertelorism	----	Optic nerve anomaly	----
11-1/pt. 1	[2,3]	Not specified	Hypertelorism, palpebral ptosis	----	----	----
ID 1	[6]	OD: posterior collar; OS: pulverulent	Downslanting palpebral fissures	----	----	Macular hypoplasia OU
ID 2	[6]	OD: dense nuclear with posterior lenticonus; OS: posterior lenticonus	----	----	Optic nerve swelling OU	Macular hypoplasia OU: avascular retina with neovascularization OU
ICN-ICW	[3,7]	Not specified	Hypertelorism	----	----	----
CSA108.01	[8]	OD: nuclear and posterior polar; OS: mild posterior polar oil droplet	Downslanting palpebral fissures	Yes	----	----
CSA108.02	[8]	Not specified	Downslanting palpebral fissures	----	----	----
Subject 2	[5]	Not specified	Hypertelorism	----	----	----
Subject 3	[5]	N/A	----	----	Optic nerve anomaly OU	Retinal hemorrhages in infancy OU
14-1/pt. 2	[3,9]	Not specified	Epicanthal folds, palpebral ptosis, esotropia	----	Optic nerve pallor	----
10-1	[3]	Not specified	Upslanting palpebral fissures, epicanthal folds	----	----	----
Individual 4	[4]	Not specified	----	----	Optic nerve anomaly	----
962112	[3]	Not specified	Upslanting palpebral fissures	----	----	----
Patient 3	[10]	N/A	----	----	----	----
Subject 1	[5]	Not specified	Hypertelorism	----	----	----
Subject 4	[5]	Not specified	Downslanting, short and narrow palpebral fissures	----	----	----
Individual 2	[4]	Not specified	Hypertelorism	----	----	----
4-1/pt. 2	[2,3]	Bilateral dense zonular central	Upslanting palpebral fissures, palpebral ptosis	----	----	----
Proband	[11]	Not specified	Bitemporal narrowing, supraorbital ridges	Yes	----	----
Mother	[11]	Mild unilateral PSC	----	----	----	----

Abbreviations: TD = Transactivation Domain; OD = Right Eye; OS = Left Eye; N/A = Not Applicable; PSC = Posterior Subcapsular Cataract, ---- = not described.

## Data Availability

The original contributions presented in the study are included in the article, further inquiries can be directed to the corresponding author.
